# Broiler stunning in electrical water bath: Validation of an animal welfare–based method for assessing stunning effectiveness

**DOI:** 10.1016/j.psj.2026.107240

**Published:** 2026-06-09

**Authors:** Yukari Togami, Jolien Hacker, Elke Rauch, Michael Erhard, Paul Schmidt, Helen Louton

**Affiliations:** a Chair of Animal Welfare, Ethology, Animal Hygiene and Animal Husbandry, Department of Veterinary Sciences, Faculty of Veterinary Medicine, LMU Munich, Veterinaerstr. 13/R, D-80539 Munich, Germany; b Animal Health and Animal Welfare, Faculty of Agriculture, Civil and Environmental Engineering, University of Rostock, Justus-von-Liebig-Weg 6b, D-18059 Rostock, Germany; c Paul Schmidt, Statistical Consulting for Science and Research, Charlottenburger Str. 77, D-13086 Berlin, Germany

**Keywords:** Poultry slaughter, Pre-slaughter handling, Electrical parameter, Consciousness, Welfare indicator

## Abstract

Electrical water bath stunning remains the predominant method for poultry slaughter in Europe, yet its welfare implications are still debated. This study assessed the current status of stunning effectiveness in broiler chickens under commercial conditions, providing a foundation for future comparisons with controlled atmosphere stunning.

Behavioral observations were made in 1 slaughterhouse over 2 study years (2021 and 2022) and were analyzed separately for each year across broilers from 4 fattening methods (**LIT**: light conventional fattening method, **HEV**: heavy conventional fattening method, **LBL**: label fattening method, **ORG**: organic fattening method). The analysis aimed to explore whether stunning performance differs according to fattening method, animal age, transport duration, carcass weight, weather conditions, and total electrical current in the electrical water bath. Stunning effectiveness was evaluated in 3 phases: pre-stunning (shackling), during stunning, and post-stunning. In each phase, key behavioral indicators, such as signs of inadequate stunning (e.g., wing flapping, body movements, rhythmic breathing), were systematically recorded. The results showed that pre-stunning behavior varied among fattening methods in 2022 (defensive reactions: ORG [0.82%] vs. LBL [0.05%]; wing flapping: LIT [29.00%] vs. OGR [17.00%], HEV [21.00%], and LBL [12.60%]), with older and lighter animals showing more pronounced defensive or flapping responses. After stunning in 2021, only ORG broilers showed cases of inadequate stunning (0.01%), whereas no incidences were observed in LIT (0.00%) and HEV broilers (0.00%). These findings highlight that even minor differences in handling and animal characteristics can significantly affect stunning effectiveness and animal welfare.

## Introduction

Electrical water bath (**EWB**) and CO_2_ stunning are currently the most widely applied methods to stun broiler chickens before slaughter in Europe (Berg and Raj, 2015; Rucinque et al., 2024). However, EWB stunning has increasingly come under scrutiny, particularly with regard to animal welfare. Several studies highlight major concerns, including stress and injuries from pre-stun handling, variability in stunning effectiveness, and the risk of animals being slaughtered while still conscious (Shields and Raj, 2010; EFSA, 2012). A central issue is the requirement to shackle the animals upside down while they are conscious, a procedure associated with pain and distress (Gentle and Tilston, 2000). Additional factors such as body size differences can reduce current delivery, leading to pre-stun shocks and incomplete loss of consciousness (Berg and Raj, 2015). Pre-stun handling alone elevates corticosterone levels, indicating considerable physiological stress before electrical exposure (Raj and Gregory, 1995). Recent research has further highlighted the welfare relevance of electrical water bath stunning and the importance of behavioral indicators for assessing stunning effectiveness under commercial slaughter conditions (Ham et al., 2025; Lin et al., 2026). These findings underline the need for continuous evaluation of pre-stunning handling and stunning procedures to improve animal welfare during poultry slaughter. Stunning efficacy is further influenced by several technical parameters—namely conductivity, voltage, frequency, exposure time, and animal size—where suboptimal settings may lead to inadequate stunning in approximately 5% to 15% of broiler chickens.

Beyond animal welfare concerns, EWB stunning is linked to carcass damage such as fractures, hemorrhages, and muscle discoloration, which reduces meat quality and marketability (Sirri et al., 2017). Acute pain responses - such as wing flapping and vigorous struggling, often induced by pre-stun shocks or inadequate restraint - further intensify stress levels and contribute to the occurrence of carcass defects (Berg and Raj, 2015). Although technical refinements, such as the use of high-frequency electrical currents, have been investigated to enhance the uniformity of stunning and minimize pre-stun shocks, achieving consistent improvements in animal welfare remains a significant challenge. Animal welfare assessment relies on both behavioral and physiological indicators. Signs such as wing flapping, vocalizations, the presence of corneal reflexes following stunning are indicative of inadequate stunning effectiveness (Shields and Raj, 2010), while blood parameters such as corticosterone and lactate serve as additional indicators of physiological stress. Comparative studies consistently show higher stress markers under EWB than under controlled CO_2_ stunning (Pinto et al., 2016; EFSA, 2012). The economic implications are also substantial as carcasses exhibiting fractures or hemorrhages are frequently downgraded or rejected, leading to direct financial losses (Sirri et al., 2017). Thus, both welfare and quality concerns highlight the need for improvements in equipment design, control of electrical parameters (e.g., voltage, amperage, and duration), and humane handling procedures.

In this context, a systematic evaluation of EWB stunning under commercial conditions is required. It is based on data from the CasStunn project (Implementation of a controlled atmosphere stunning as an alternative to an electrical bath stun for broilers), which assessed both stunning effectiveness and carcass quality outcomes during the 2021/2022 period. The study specifically aims to compare four common broiler fattening methods across three husbandry systems in Germany, including conventional broilers (heavy and light fattening) in husbandry system 2, broilers in husbandry system 3, and organic broilers in husbandry system 4. More precisely, its objectives are to evaluate the effectiveness of EWB stunning, aiming to identify its main strengths and limitations across husbandry systems, explore improvement opportunities, and contribute to the development of more animal-friendly slaughter practices.

## Animals, materials, and methods

### Animals, fattening methods, and husbandry systems

To investigate the procedure, we examined male and female broiler chickens from mixed flocks from various farms by observing their behaviors at the slaughterhouse. The animals for the study were selected through a randomization process applied at a poultry slaughterhouse in southern Germany. The study included four distinct fattening methods (**LIT**: Light conventional fattening method, **HEV**: Heavy conventional fattening method, **LBL**: label fattening method, **ORG**: organic fattening method) within 3 husbandry systems, as defined by the Haltungsform.de (2023) (Initiative Tierwohl GmbH, Bonn, Germany). Husbandry system 1 was not included in this study, as no corresponding flocks were available during the sampling period. For the purpose of this investigation conducted in 2021 and 2022, data were collected from flocks representing: (i) light and heavy conventional fattening methods (LIT and HEV, both classified as husbandry system 2), (ii) a label fattening method (LBL, husbandry system 3), and (iii) an organic fattening method (ORG: husbandry system 4). The husbandry systems are defined by several criteria, including stocking density and genotype, and differed in their maximum stocking densities and broiler genotypes (Table 1). In total, 62 flocks were examined, including 16 flocks with 212,000 Ross 308 broilers (**LIT**), 18 flocks with 243,300 Ross 308 broilers (**HEV**), 13 flocks with 133,850 Ranger Classic broilers (**LBL**), and 15 flocks with 103,800 Hubbard JA 747 broilers (**ORG**). Flock sizes varied considerably across fattening methods, ranging from approximately 4,800 to 20,000 broiler chickens per flock.

**Table 1:** Overview of husbandry systems and defining criteria for broiler chickens. This table summarizes the main characteristics of the 4 husbandry systems for broiler chickens as defined by Haltungsform.de in Germany. In the study fattening methods were separated in LIT = light conventional fattening method, HEV = heavy conventional fattening method, LBL = label fattening method, and ORG = organic fattening method. Husbandry system 1 was not investigated in the present study, whereas husbandry system 2 was differentiated into light and heavy fattening methods. The systems differ in maximum stocking density, housing and environmental conditions, available enrichment materials, and the genetic lines used. The minimum requirements for standard barn housing are defined by the German Ordinance on the Welfare of Farm Animals (Tierschutz-Nutztierhaltungsverordnung, TierSchNutztV, 2001).

Criterion	Husbandry system 1: Indoor housing	Husbandry system 2: Indoor + extra space	Husbandry system 3: Outdoor climate access	Husbandry system 4: Free-range (Premium)
**Fattening methods in the study**	Not assessed	LIT and HEV	LBL	ORG
**Maximum stocking density**	Up to 39 kg/m^2^	Up to 35 kg/m^2^	Up to 25 kg/m^2^ or a maximum of 29 kg/m^2^ in barns with an outdoor climate area	Up to 21 kg/m^2^
**Housing and environment**	Standard barn housing without outdoor access	Standard barn housing without outdoor access	Barns with permanent access to an outdoor climate area	Barns with access to a vegetated outdoor area for ≥ one-third of lifetime; the area must be predominantly covered with vegetation; structural elements must provide shelter for the animals
**Enrichment materials**	Dry litter suitable for pecking, scratching, and dustbathing	Organic enrichment (e.g., straw bales, pecking stones); at least 1 enrichment object for every 150 m^2^	Organic enrichment (e.g., straw bales or pecking stones); at least 2 objects per 150 m^2^ or 3 straw/hay bales per 2,000 broilers and 1 pecking object per 1,000 broilers	Additional litter (straw, wood shavings, sand, or peat) on at least one-third of the barn area
**Breed (genetic line)**	Generally robust and healthy breeds	Generally robust and healthy breeds	Generally robust and healthy breeds; slow-growing breeds (≤45 g/day; ≤51 g/day with gait score test) or fast-growing breeds with a minimum slaughter age of 81 days	Generally robust and healthy breeds; slow-growing breeds (≤45 g/day) or fast-growing breeds slaughtered at ≥81 days

### Slaughterhouse and assessment positions

**Fig. 1:**
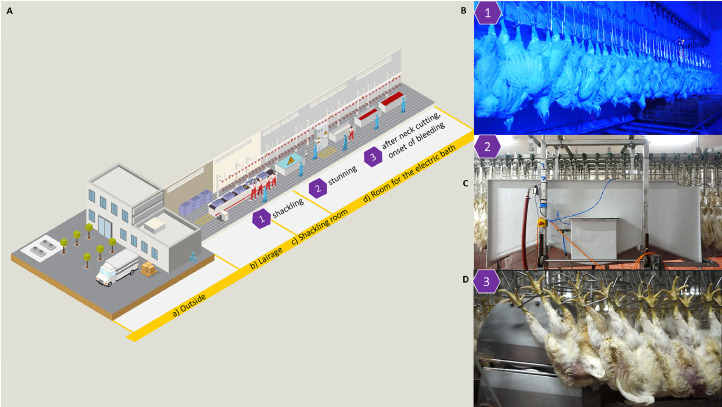
(A) The 3 data collection points within the slaughterhouse, indicated by violet numbers on the map. The yellow line indicates the 4 measurement locations for temperature and relative humidity. (B) Animals after shackling and before stunning. (C) Electrical water bath. (D) Animals after neck cutting.

Data collection took place in a commercial poultry slaughterhouse during 2 periods: from May to October 2021 and from June to August 2022. The interruption between these periods was due to project-related scheduling and operational organization of the data collection phase, during which no data collection could be performed. The duration of the slaughter process varied depending on the flock size, ranging from approximately 1 hour for flocks of around 4,800 animals to approximately 4 hours for flocks of around 20,000 animals. The parameters were assessed in at least 3 observational periods (**OP**) for each flock, covering the beginning, middle, and end of the slaughter process. The broilers were assessed at 3 distinct stages: (1) pre-stunning during shackling, (2) during stunning, and (3) immediately after stunning, at the onset of bleeding. The specific positions where each parameter was recorded are illustrated in Fig. 1A in purple, and the assessed parameters are shown in Table 2. All investigations were done by the same 2 observers.

**Table 2:** Animal behavior index with assessment positions across 3 stages of the slaughter process, with each observed parameter, detailed definition, number of recorded cases, and corresponding scores during the observational period (OP).

Position	Parameter or behavior	Definition	Time or animals per OP	Score
**1. Shackling**	Defensive reaction	Moving away, pecking, attempting to escape from staff during shackling	20 Animals	Number of cases
	Wing flapping	Multiple flapping with one or both wings	20 Animals	Number of cases
**2. Stunning**	Pre-stun electric shocks	Sudden wing movements and signs of resistance to the water bath	1 minute	Number of cases
**3. After neck cutting**	Wing flapping	Multiple flapping with one or both wings	1 minute	Number of cases
	Body movements	Any body movement, lifting of the head, directed gaze, blinking	1 minute	Number of cases
	Rhythmic breathing	Regular breathing with at least 2 breath movements of the pelvic floor or abdomen	1 minute	Number of cases
	Corneal reflex	Involuntary blinking of the eyelids after touching the cornea	5 Animals	Number of cases
	Pupillary reflex	Pupils become smaller after being flashed with a flashlight	5 Animals	Number of cases
	Signs of inadequate stunning	The animal shows distinct indications of consciousness, such as raising the head	1 minute	Number of cases

### General data

We collected comprehensive slaughter-related data for the flocks, including the animals’ age (in days), transport duration from the end of farm loading to arrival at the slaughterhouse (in hours and minutes), and mean carcass weight (in kilograms). Carcass weight, which generally accounts for approximately 70% to 75% of live weight after removal of feathers, viscera, and blood, was used as a proxy for live weight in this study (Leeson and Summers, 2001). In addition, temperature (°C) and relative humidity (%) were recorded at 5-minute intervals by using data loggers (LOG 210, Dostmann electronic GmbH, Wertheim, Germany). These loggers were strategically placed at 4 distinct locations throughout the entire slaughter process: a) outside the lairage, b) within the lairage area, c) in the room for shackling the animals, and d) in the room for the EWB, neck cutting, bleed-out, and scalding processes (Fig. 1A). Explanatory variables were selected a priori based on their biological relevance and availability under commercial slaughterhouse conditions. Only routinely obtainable, non-invasive parameters were included to avoid interference with standard processing procedures.

### Key parameters of the slaughter process

The key parameters included the mean time elapsed between shackling the animals on metal shackles and their immersion into the EWB (in seconds and milliseconds), the time between stunning and neck cutting (stun-to-stick time in seconds and milliseconds), and the total bleeding duration (in minutes and seconds). Key parameters related to stunning were documented, analog to the other measurements with a minimum of 1 recording per slaughter process for each flock.

### Electrical water bath

For the stunning of the broilers, an EWB (Lebensmitteltechnik-Anlagenbau GmbH, Nienburg, Germany) with a constant current stunner with pulsed alternating current was routinely used in the slaughterhouse for standard operations (Fig. 1C). This water bath measures 2.15 meters in length and 0.50 meters at its lowest point, allowing for the simultaneous stunning of 5 broilers, with each animal being exposed to the water for a duration of 5 seconds. The neck cutting after the stunning is automated, and the height of the water bath is adjusted by the staff at the beginning of the slaughter of each flock, according to the size of the animals, to ensure proper contact with the water and effective stunning. The electrical parameters of the water bath, including voltage in Volt, total current in Milliampere, and frequency in Hertz, were constantly measured per flock and day with a DSM 96 digital measuring device (Müller + Ziegler GmbH, Gunzenhausen, Germany). The data were stored electronically as an average per minute by using a JUMO LOGOSCREEN 500 cf recorder (JUMO GmbH und Co. KG, Fulda, Germany). The resulting data is available only at the flock level, not at the level of individual animals. Furthermore, these parameters were monitored from the control panel during a minimum of 3 OP.

### Assessment of pre-stunning indicators during shackling

Pre-stunning indicators were evaluated by quantifying the mean frequencies of defensive reactions and wing flapping observed during shackling. The broilers from the different farms were initially transported from the lairage area to the entrance of the shackling room by using transport containers equipped with plastic crates. While the broilers were being hung (number 1 in Fig. 1A and 1B), 2 to 4 employees from the slaughterhouse were positioned next to each other. The broilers were manually removed from the crates and suspended head down by their legs on shackles that moved to the right. The lighting in the room varied frequently, ranging from slightly dimmed to blue light, based on the task and experience of employees. Behavioral observations were conducted directly on-site by the same trained observer throughout the study. Throughout the shackling process, defensive reactions during handling before shackling were assessed (Table 2), including behaviors such as moving away from staff, pecking, avoidance, or attempting to escape during handling and shackling. Wing flapping was subsequently monitored after the broilers had been fully shackled, recorded at the moment when the staff had no further physical contact with the broilers (Table 2). Wing flapping was defined as repeated flapping movements involving one or both wings after release by the handler. These assessments were made at each employee position along the shackling line: the beginning, middle position 1, middle position 2, and the position closest to the EWB. Twenty animals per OP were assessed to determine the presence or absence of these behaviors.

### Assessment of behavior during stunning

While the broilers were immersed in the water bath (number 2 in Fig. 1A and 1C), those that had received pre-stun electric shocks were counted over a 1-minute period as they passed along the moving slaughter line (Table 2). Pre-stun electric shocks were identified based on defensive reactions, primarily wing flapping and abrupt body movements occurring before proper head contact with the electrified water. These reactions were used as indicators of unintentional electrical stimulation outside the intended stunning process.

### Assessment of stunning effectiveness after stunning

The assessment of stunning effectiveness was conducted separately to distinguish between signs of inadequate stunning and those clearly associated with inadequate stunning, with the former referring to subtle indicators that may suggest ineffective stunning, and the latter indicating unmistakable evidence of a failed stun. To assess the effectiveness of stunning after immersion in the EWB, behaviors indicative of inadequate stunning such as wing flapping, body movements, and rhythmic breathing were assessed approximately 15 seconds after the neck cutting (number 3 in Fig. 1A and 1D). Observers were stationed at each point for a duration of 1 minute, enabling the assessment of 80 animals per position and OP. Furthermore, in 5 animals, the pupillary light reflex, assessed using a flashlight, and the corneal reflex, evaluated by gently touching the cornea with a finger, were examined (Moore et al., 2022) (Table 2). At the same position, animals were monitored for 2 minutes for signs of consciousness, such as head raising with eyes open and blinking, which could indicate potentially ineffective stunning (Table 2).

### Interobserver reliability test

Before the main data collection, an interobserver reliability test was conducted involving 3 veterinarians. Interobserver agreement was assessed using the prevalence-adjusted and bias-adjusted kappa (**PABAK**) method, following the approach described by Byrt et al. (1993). At each observation point, the behavior of between 15 and 400 Ross 308 broilers was evaluated. The observed behavioral reactions were converted into percentages and descriptively analyzed. For the subsequent data collection, behavioral assessments were performed by 2 trained veterinarians with prior experience in animal welfare research. The PABAK coefficient was calculated as: PABAK = (*k* × *p₀* – 1) / (*k* – 1), where *k* is the number of categories and *p₀* is the proportion of agreement between observers.

### Statistical analysis

All analyses were performed in R (R Core Team, 2025; Vienna, Austria). Outcomes included behavior during shackling, number of inadequately stunned animals, and frequency of pre-stun shocks. Environmental conditions on slaughter days (temperature and relative humidity) and all explanatory variables (fattening method, age, transport duration, carcass weight, weather conditions, and total electrical current in the EWB) were descriptively summarized. Associations between explanatory variables and outcomes were assessed using negative binomial regression models, which are appropriate for overdispersed count data with excess zeros. These methods were considered more appropriate for the structure and distribution of the present dataset. Rate ratios (RR) and corresponding 95% confidence intervals (CI) denote exponentiated coefficients from the negative binomial regression models and quantify multiplicative differences in expected counts between groups; they are used here as model-based effect estimates (i.e., count ratios) and do not represent epidemiological incidence rate ratios derived from exposure–control comparisons. A stepwise modeling approach was applied: Model 1 included only fattening method; model 2 additionally adjusted for age and carcass weight; and model 3 further controlled for total electrical current. Model 2 was selected for primary reporting because it provided the best balance of model fit (Akaike information criterion) and interpretability while avoiding potential overadjustment. Model adequacy was evaluated using residual diagnostics, Akaike information criterion, and nested model comparisons. For continuous outcomes (e.g., test measurements across fattening methods or fattening durations), linear mixed-effects models were used with slaughter day as a random intercept to account for clustering. In addition to the primary analyses using negative binomial regression models, predefined subgroup analyses were conducted to explore whether the observed effects varied across key explanatory variables. These variables included fattening method, animal age, transport duration, carcass weight, weather conditions, and total electrical current in the EWB. These subgroup analyses were intended to provide a more detailed interpretation of the main findings and to assess the robustness of the observed effects across different model specifications.

## Results

The PABAK value of interobserver reliability ranged from 0.83 to 1.00, with a mean of 0.97, indicating excellent agreement between observers across all evaluated behavioral parameters.

The general data, including fattening method, animal age, and transport duration from farm loading to slaughterhouse arrival, mean carcass weight, ambient temperature, and relative humidity, are presented in Table 3.

**Table 3:** Summary of general data for each year and for the combined 2-year period. Data are presented separately for each fattening method: LIT: light conventional fattening method, HEV: heavy conventional fattening method, LBL: label fattening method, ORG: organic fattening method. The table allows comparison of annual and cumulative results across fattening methods. Number of observations in parentheses.

Parameter	Year	LIT	HEV	LBL	ORG	Total
**Number of flocks**	2021	13	15	–	12	40
	2022	3	3	13	3	22
	total	16	18	13	15	62
**Mean age (days)**	2021	32.27 (77)	39.70 (88)	–	55.29 (70)	41.91 (235)
	2022	33.33 (18)	40.33 (18)	43.30 (60)	64.38 (13)	43.68 (49)
	total	32.47 (95)	39.81 (106)	43.30 (60)	56.71 (83)	42.21 (284)
**Mean transport duration (minutes)**	2021	33.51 (77)	31.26 (88)	–	134.64 (70)	62.79 (235)
	2022	16.00 (18)	34.67 (18)	99.62 (60)	234.92 (13)	91.22 (49)
	total	30.19 (95)	31.84 (106)	99.62 (60)	150.35 (83)	67.79 (284)
**Mean carcass weight (kg)**	2021	1.23 (77)	1.82 (82)	–	1.63 (70)	1.56 (229)
	2022	1.17 (18)	1.72 (18)	1.67 (60)	1.76 (13)	1.61 (49)
	total	1.22 (95)	1.80 (100)	1.67 (60)	1.65 (83)	1.57 (278)
**Mean temperature outside (**°**C)**	2021	14.53 (77)	15.79 (88)	–	14.67 (70)	15.04 (235)
	2022	17.20 (18)	19.50 (18)	18.48 (60)	18.31 (13)	18.42 (49)
	total	15.04 (95)	16.42 (106)	18.48 (60)	15.24 (83)	15.63 (284)
**Mean relative humidity outside (%)**	2021	77.92 (77)	74.68 (88)	–	72.66 (70)	75.14 (235)
	2022	66.60 (12)	58.63 (18)	71.05 (56)	81.53 (9)	69.13 (39)
	total	76.39 (89)	71.95 (106)	71.05 (56)	73.67 (79)	74.28 (274)
**Mean temperature lairage (**°**C)**	2021	16.32 (77)	17.06 (88)	–	16.05 (70)	16.52 (235)
	2022	18.40 (18)	18.87 (18)	18.67 (60)	18.33 (13)	18.62 (49)
	total	16.71 (95)	17.37 (106)	18.67 (60)	16.41 (83)	16.88 (284)
**Mean relative humidity lairage (%)**	2021	80.64 (77)	81.44 (88)	–	82.29 (70)	81.43 (235)
	2022	76.53 (18)	71.00 (18)	77.46 (60)	84.75 (13)	77.11 (49)
	total	79.86 (95)	79.67 (106)	77.46 (60)	82.68 (83)	80.69 (284)
**Mean temperature shackling room (**°**C)**	2021	22.78 (77)	23.60 (88)	–	23.49 (70)	23.30 (235)
	2022	24.90 (18)	25.30 (18)	24.80 (60)	24.78 (13)	24.90 (49)
	total	23.18 (95)	23.89 (106)	24.80 (60)	23.69 (83)	23.57 (284)
**Mean relative humidity shackling room (%)**	2021	78.51 (77)	76.98 (88)	–	72.43 (70)	76.13 (235)
	2022	74.63 (18)	68.20 (18)	73.57 (60)	76.55 (13)	73.21 (49)
	total	77.77 (95)	75.49 (106)	73.57 (60)	73.08 (83)	75.62 (284)
**Mean temperature slaughter room (**°**C)**	2021	27.62 (77)	28.05 (88)	–	27.71 (70)	27.81 (235)
	2022	28.13 (18)	28.40 (18)	28.16 (60)	27.92 (13)	28.17 (49)
	total	27.72 (95)	28.11 (106)	28.16 (60)	27.74 (83)	27.87 (284)
**Mean relative humidity slaughter room (%)**	2021	86.27 (77)	84.76 (88)	–	85.12 (70)	85.36 (235)
	2022	83.60 (18)	79.77 (18)	83.98 (60)	88.28 (13)	83.73 (49)
	total	85.76 (95)	83.91 (106)	83.98 (60)	85.61 (83)	85.08 (284)

Regarding key parameters of the slaughterhouse, the mean time between shackling and immersion in the electrical water bath (EWB) was 50.09 seconds in 2021 (n = 1.224) and increased slightly to 52.05 seconds in 2022 (n = 585). The interval between stunning and neck cutting (stun-to-stick time) was 9.96 seconds in 2021 (n = 234) and 10.41 seconds in 2022 (n = 49). The mean total bleeding duration increased from 3 minutes and 53 seconds in 2021 (n = 118) to 4 minutes and 5 seconds in 2022 (n = 49). The electrical parameters of the water bath, including voltage (V), total current (mA), and frequency (Hz), are provided in Table 4. Voltage levels in the EWB were higher in ORG compared to the other fattening methods.

**Table 4:** Mean electrical parameters of water bath stunning, presented for each year and for the combined 2-year period. Data are shown separately for each fattening method: LIT: light conventional fattening method, HEV: heavy conventional fattening method, LBL: label fattening method, and ORG: organic fattening method, Number of observations in parentheses.

Parameter	Year	LIT	HEV	LBL	ORG	Total
**Voltage (V)**	2021	145.61 (66)	126.55 (66)	–	178.33 (70)	150.72 (202)
	2022	119.72 (18)	121.98 (18)	147.39 (60)	203.18 (13)	145.28 (49)
	total	140.06 (84)	125.57 (84)	147.39 (60)	182.22 (83)	149.66 (251)
**Total current (mA)**	2021	920 (66)	860 (66)	–	880 (70)	890 (202)
	2022	1,100 (18)	1,130 (18)	1,200 (60)	1,380 (13)	1,200 (49)
	total	960 (84)	920 (84)	1,200 (60)	960 (83)	950 (251)
**Frequency (Hz)**	2021	50.91 (66)	50.88 (66)	–	50.90 (70)	50.90 (202)
	2022	50.64 (18)	50.91 (18)	50.94 (60)	50.90 (13)	50.88 (49)
	total	50.85 (84)	50.89 (84)	50.94 (60)	50.90 (83)	50.89 (251)

### Assessment of pre-stunning indicators during shackling

#### Mean frequency of defensive reactions

The occurrence of defensive reactions during shackling was generally low across all fattening methods and study years. The analysis revealed a significantly higher risk of defensive reactions in ORG broilers compared to HEV broilers in 2021 (RR = 3.06, 95% CI: 1.08–8.61; P = 0.035). In 2022, ORG broilers showed a higher risk compared to LBL broilers (RR = 24.09, 95% CI: 1.36–427.52; P = 0.030). In addition, older animals were more likely to show defensive reactions compared to younger animals in 2021 (RR = 2.25, 95% CI: 1.04–4.87; P = 0.040). Table 5 shows mean descriptive results of defensive reactions. Due to the overall low incidence of defensive reactions, relative risk estimates should be interpreted in the context of low absolute event frequencies. No statistically significant association with carcass weights was detected.

**Table 5:** Mean percentage (± SD) of broilers displaying each behavioral parameter out of all assessed broilers, presented separately for each year and for the combined 2-year period. Data are shown separately for each fattening method: LIT = light conventional fattening method, HEV = heavy conventional fattening method, LBL = label fattening method, and ORG = organic fattening method.

Parameter	Year	LIT	HEV	LBL	ORG
**Defensive reaction (%)**	2021	0.23 (1.14)	0.27 (1.35)	–	1.18 (3.98)
	2022	0.07 (0.60)	0.14 (0.84)	0.04 (0.47)	0.87 (2.85)
**Wing flapping (%)**	2021	48.40 (29.43)	41.56 (32.70)	–	28.18 (20.70)
	2022	28.70 (24.34)	20.64 (24.98)	16.83 (18.83)	16.70 (16.66)
**Pre-stun electric shocks (%)**	2021	0.01 (0.01)	0.01 (0.01)	–	0.00 (0.01)
	2022	0.00 (0.01)	0.01 (0.01)	0.00 (0.01)	0.00 (0.01)
**Wing flapping (%)**	2021	0.00 (0.00)	0.00 (0.00)	–	0.00 (0.01)
	2022	0.00 (0.00)	0.00 (0.01)	0.00 (0.01)	0.00 (0.01)
**Body movements (%)**	2021	0.01 (0.01)	0.01 (0.01)	–	0.01 (0.01)
	2022	0.04 (0.04)	0.04 (0.03)	0.03 (0.03)	0.01 (0.01)
**Rhythmic breathing (%)**	2021	0.01 (0.02)	0.02 (0.02)	–	0.02 (0.03)
	2022	0.01 (0.01)	0.01 (0.01)	0.01 (0.01)	0.01 (0.01)
**Pupillary reflex (%)**	2021	0.02 (0.06)	0.05 (0.10)	–	0.04 (0.11)
	2022	0.02 (0.06)	0.10 (0.14)	0.07 (0.16)	0.00 (0.00)
**Corneal reflex (%)**	2021	0.02 (0.07)	0.04 (0.09)	–	0.05 (0.13)
	2022	0.04 (0.09)	0.09 (0.17)	0.05 (0.12)	0.02 (0.06)
**Inadequate stunning (%)**	2021	0.00 (0.00)	0.00 (0.00)	–	0.01 (0.01)
	2022	0.00 (0.01)	0.00 (0.01)	0.01 (0.01)	0.01 (0.01)

#### Mean frequency of wing flapping

During EWB stunning, broilers are suspended on the slaughter line while still conscious. Broilers from LIT and HEV showed wing flapping during shackling more frequently than those from LBL and ORG. Overall, a general reduction in this behavior was observed from 2021 to 2022 across all fattening methods. The descriptive presentation of the results is presented in Table 5. In 2022, wing flapping was more frequent in LIT broilers than in ORG (RR = 1.84, CI: [1.31, 2.57], *P* < 0.001), HEV (RR = 1.38, CI: [1.15, 1.65], *P* < 0.001), and LBL broilers (RR = 1.91, CI: [1.54, 2.38], *P* < 0.001). Carcass weight was significantly associated with wing flapping during shackling, with higher carcass weight associated with a reduced risk of wing flapping in both 2021 (RR = 0.79, CI: [0.72, 0.87], *P* < 0.001) and 2022 (RR = 0.88, CI: [0.82, 0.95], *P* < 0.001).

### Assessment of behavior during stunning

The incidence of pre-stun electric shocks was minimal or negligible in broilers from all 4 fattening methods. No significant association between fattening method and the occurrence of pre-stun electric shocks was observed. The effect of age was statistically significant, with older broilers showing a higher rate of pre-stun electric shocks than younger ones (RR: 1.37, CI: [1.02, 1.85], *P* = 0.036) in 2021.

### Assessment of stunning effectiveness after stunning

#### Signs of inadequate stunning

In 2021, ORG broilers showed a higher rate of wing flapping than LIT (RR: 3.45, CI: [1.21, 9.83], *P* = 0.021) and HEV broilers (RR: 5.39, CI: [1.65, 17.59], *P* = 0.005). The effect of total current was statistically significant, with higher voltage associated with less wing flapping in 2021 (RR: 0.49, CI: [0.33, 0.73], *P* < 0.001). In 2022, differences in wing flapping between fattening methods were not statistically supported, and no subgroup analyses were conducted for this year. Body movement rates were very low in both years, with no differences between fattening methods. The effect of total current was statistically significant, with higher voltage associated with fewer body movements in both 2021 (RR: 0.74, CI: [0.57, 0.97], *P* = 0.027) and 2022 (RR: 0.60, CI: [0.50, 0.71], *P* < 0.001). In both years, the differences in rhythmic breathing between fattening methods were not statistically significant. The effect of total current was statistically significant, with higher voltage associated with fewer cases of rhythmic breathing in 2021 (RR: 0.76, CI: [0.66, 0.89], *P* < 0.001). In 2021, ORG broilers showed a higher rate of pupillary reflex than LIT broilers (RR: 3.28, CI: [1.15, 9.33], *P* = 0.026). A higher total current in 2021 was associated with a reduced occurrence of pupillary reflex (RR: 0.67, CI: [0.45, 0.98], *P* = 0.041). For the corneal reflex, we found no significant differences between fattening methods in either year, whereas age had an effect in 2022, with older broilers showing a higher rate of corneal reflex than younger ones (RR: 3.68, CI: [1.41, 9.58], *P* = 0.008).

#### Inadequate stunning observed after neck cutting

In 2021, inadequate stunning occurred more often in ORG broilers than in both LIT (RR: 3.31, CI: [1.60, 6.86], *P* < 0.001) and HEV broilers (RR: 3.06, CI: [1.48, 6.34], *P* = 0.003). A similar pattern was observed in 2022, with ORG broilers showing a higher occurrence of inadequate stunning than LIT (RR: 4.20, CI: [1.24, 14.23], *P* < 0.021) and HEV broilers (RR: 3.48, CI: [1.10, 10.96], *P* < 0.033). In addition, age played a role: In 2021, older animals showed a lower likelihood of inadequate stunning than younger ones (RR: 0.74, CI: [0.63, 0.87], *P* < 0.001).

## Discussion

The present study demonstrates that pre-stunning behavior during shackling varies according to fattening method, with older animals showing more pronounced defensive reactions and lighter animals showing increased wing flapping. Notably, inadequate stunning occurred in 2021 only in ORG broilers, whereas no such cases were observed in LIT and HEV broilers. These results indicate that even subtle differences in handling procedures and animal characteristics can significantly affect stunning effectiveness and overall animal welfare.

Pre-stunning handling procedures, such as shackling, are known to elicit stress and defensive reactions in broilers due to physical restraint and sensory stimulation, which may compromise animal welfare and affect stunning effectiveness. Against this background, the present study aimed to evaluate how fattening methods, age, and related animal- and process-specific factors influence pre-stunning behavior and stunning outcomes under commercial slaughter conditions.

### Assessed animal parameters

The mean time between shackling the animals on metal shackles and their immersion into the EWB complied with the maximum duration of 60 seconds as stipulated by Council Regulation (EC) No 1099/2009 (Annex II, Section 5.2). This finding indicates that, in terms of timing, this part of the stunning process met the minimum legal standards for the protection of animals. Regarding the mean time interval between stunning and neck cutting (stun-to-stick time), the German Animal Welfare Slaughter Ordinance (Tierschutz-Schlachtverordnung, TierSchlV, 2012) defines a maximum duration of 20 seconds and requires bleeding to be conducted while the animal is suspended (bleeding in shackling position) to ensure rapid and complete blood loss. The difference of 12 seconds in the mean total bleeding duration between 2021 and 2022 was due to slight modifications inside the slaughterhouse and an extension of the bleeding line. Although the Council Regulation does not specify an exact minimum bleeding duration after EWB stunning, it requires that bleeding commence as quickly as possible and before consciousness returns (Council Regulation [EC] No 1099/2009, Annex II, water bath section). It further states that animals must remain unconscious until death, implying that bleeding must be sufficiently long and effective to ensure unconsciousness, as confirmed by the results of our study. During stunning with an EWB, according to Council Regulation (EC) No 1099/2009 (Annex I, Section 6.3), animals must be exposed to a minimum current of at least 100 mA (<200 Hz), 150 mA (200 to 400 Hz), or 200 mA (400 to 1,500 Hz) for a minimum duration of 4 seconds per animal. Voltage and frequency settings must ensure these current levels; however, no specific voltage values are mandated. All 3 current values used in this study fell within the specified range on average. Individual animal parameters could not be collected.

On average, ORG broilers showed defensive reactions during shackling more frequently than broilers of the other fattening methods. The differences between ORG and HEV were statistically significant in 2021. ORG broilers also showed increased defensive reactions toward the end of the shackling line (position closest to the EWB). This may partly be because less experienced staff members (≤6 months of experience) were often assigned to positions near the end of the line, where their animal handling may have been less confident than that by experienced employees (>6 months of experience). Furthermore, as fewer animals remain in the crates toward the end, the calming effect of flock proximity decreases, and the animals become less calm. Based on findings by Wessel et al. (2022), carrying broilers together with several neighboring animals may reduce wing flapping, due to increased physical stabilization and reduced agitation. Close physical contact between neighboring birds may mechanically limit excessive movement and provide support during handling, thereby reducing imbalance, agitation, and escape-related wing movements. In addition, Wigham et al. (2019) observed that, because the number of birds per crate can vary, operators in the final position are responsible for shackling surplus animals, which often resulted in rough handling. As ORG broilers were more active, this could also be the case in our study, leading to more defensive reactions. Age was also a relevant factor in our study: Older animals showed more pronounced defensive reactions, which may be related to the organic husbandry system owing to the use of slower growing fattening breeds. In our study, ORG broilers had a mean age of 56.71 days, more than 10 days older than broilers from the other fattening methods. A study on environmental enrichment has shown that broilers in enriched environments display elevated levels of activity and agility (de Jong, 2021). Dawson et al. (2021), for example, found that slower growing breeds were more active and spent more time standing and walking than faster growing breeds, which were more inactive.

The mean frequency of wing flapping during shackling was significantly highest in LIT broilers. Furthermore, carcass weight and wing flapping were negatively correlated in that lighter animals flapped their wings more often than heavier ones. The lower body mass of LIT broilers may have reduced their stability in the shackles. In our study, the size of shackles and spacing between each shackle were not adjusted to animal size. Consequently, LIT broilers often had larger distances between individuals at the start of the shackling process, leading to reduced body contact, less physical stabilization, and increased agitation. Debut et al. (2005) showed that shackling is a stressful procedure, indicated by elevated plasma corticosterone levels. This finding, that shackling causes stress in animals, together with the findings of Wessel et al. (2022), who assessed the effects of 2 manual catching methods on injury risks and the behavior of broilers, and the present observations collectively support the idea that social buffering and calm, predictable environments can mitigate stress. Kannan et al. (1997) reported that excessive wing flapping increases the risk of bruises and fractures and may compromise carcass quality mainly through tissue damage, particularly affecting meat appearance and integrity. Effective mitigation measures include dimmed lighting in the shackling area (e.g., by using curtains). Blue light has also been shown to reduce anxiety-related behavior in poultry, thereby promoting calmer handling during pre-slaughter procedures (Remonato Franco et al., 2022). It would therefore be reasonable to use blue light consistently to maintain a calming effect on the animals. This measure was not implemented in the slaughterhouse of the present study. From an animal welfare perspective, pronounced defensive reactions and wing flapping can increase the risk of injury in birds. Pre-stunning pain, fear, and distress during shackling are not permissible under European legislation (Council Regulation [EC] No 1099/2009) and must be minimized through appropriate slaughter practices. Measures include adequate staff training, calm handling, and adjustments to minimize agitation along the shackling line. These improvements not only support compliance with legal standards but also enhance both animal welfare and meat quality throughout the slaughter process.

Pre-stun electric shocks were observed only infrequently in the present study. Across all fattening methods and both years, the pre-stun electric shocks occurred in fewer than 0.01% of cases. However, such reactions should not occur under correctly applied EWB stunning conditions and can cause considerable pain to the animals before contact with the electrified water bath (Shields and Raj, 2010). Inadequate EWB design and improper bath height are key risk factors (Berg and Raj, 2015). The bath height must be precisely adjusted to the animals’ body size to ensure full immersion of the head and, in smaller animals, the neck up to the base of the wings during stunning (World Organisation for Animal Health, 2024). Significant variation in body size within a flock may exacerbate the problem: Larger birds may enter the bath wings-first, while smaller birds may fail to achieve full contact. Our statistical analysis using negative binomial regression revealed no significant effect of fattening methods on the occurrence of pre-stun shocks. However, older broilers showed a higher incidence than younger ones, suggesting that age and potentially body size uniformity may influence the occurrence. Fuseini et al. (2023) proposed the use of a ramp leading to the EWB as a straightforward measure, which is common practice in commercial slaughterhouses. In the slaughterhouse examined in our study, such a ramp was in place, but nevertheless, unexpected pre-stun electric shocks still occurred. This finding suggests that the installation, adjustment, and maintenance of the ramp warrant closer examination to ensure optimal functionality and animal welfare compliance (Council Regulation [EC] No 1099/2009; Devos et al., 2018). Proper adjustment of equipment, regular monitoring, and personnel training to control these indicators are essential to ensure all animals are stunned effectively and without avoidable pain or distress.

The results regarding wing flapping, body movements, rhythmic breathing, and pupillary reflex after neck cutting were evaluated as potential indicators of inadequate stunning. In 2021, ORG broilers showed a significantly increased risk of wing flapping compared with LIT and HEV broilers. A clear pattern emerged: In 2021, higher total electrical current during EWB stunning was associated with a lower frequency of wing flapping, rhythmic breathing, pupillary reflex, and body movements. Wing flapping, body movements, and rhythmic breathing reflect different physiological mechanisms and time frames, and they all can indicate incomplete or shallow unconsciousness if electrical parameters are insufficient (Prinz et al., 2010). We additionally assessed the pupillary reflex as a neurological indicator. In 2021, ORG broilers showed a significantly higher rate of positive pupillary reflex than LIT broilers. Brainstem reflexes such as the pupillary and corneal reflexes indicate residual brainstem activity and potentially incomplete loss of consciousness (Raj et al., 1991). Given that reflex activity was assessed approximately 15 seconds after neck cutting, the persistence of a pupillary reflex in some animals may reflect inadequate stunning depth (Croft, 1961). Individual indicators after neck cutting in our study alone are not definitive markers of consciousness, but their combined presence provides strong evidence of ineffective stunning. In 2022, the corneal reflex was observed significantly more frequently in older than in younger broilers, suggesting a potential association between age and the potential indicators of inadequate stunning following EWB stunning. The statistical analysis indicated an association between age and the occurrence of defensive reactions based on negative binomial regression. This could be related to physiological or anatomical changes in older animals, such as altered nerve sensitivity, corneal integrity, or differences in central nervous system function (Stepp et al., 2018). Alternatively, this observation may reflect confounding factors such as fattening method, handling differences, or stunning parameters in ORG broilers. While reflex testing is an established method in routine slaughter monitoring, physiological measures such as electroencephalography could also be used for the scientific validation of unconsciousness assessment (Sandercock et al., 2014). From an animal welfare perspective, Council Regulation (EC) No 1099/2009 requires that all animals must be rendered unconscious without avoidable pain or distress, highlighting the importance of optimizing stunning parameters for older and potentially more resistant animals.

At the position after neck cutting, signs of inadequate stunning were observed only in LBL and ORG broilers, with an incidence of 0.01%. Inadequate stunning is considered the most critical aspect of stunning effectiveness, as animals may either not be stunned at all or regain consciousness after stunning. This situation constitutes a serious animal welfare concern (Raj, 1998). During the study period, statistically significant differences between ORG and LIT as well as ORG and HEV broilers were observed in 2021, indicating a higher incidence of inadequate stunning in ORG broilers (0.01% vs. 0.00%). In contrast, no differences between fattening methods were detected in 2022. These findings suggest that ORG broilers may be more difficult to stun effectively under standard EWB parameters. Animal age also played a role: Younger broilers were more likely to show signs of inadequate stunning than older ones. The apparent age effect may partly reflect confounding by fattening method. Age is associated with shifts in muscle composition and protein distribution (e.g., fiber size, water and protein content, fat infiltration), which are known to influence tissue electrical impedance or conductivity (Rutkove and Sanchez, 2019; Choi et al., 2023). Because these characteristics may be more common in certain age groups within ORG fattening method, age appeared as a significant factor in the analysis, even though it may not be the primary cause by itself.

In EWB systems, delays between stunning and neck cutting may permit recovery of consciousness, as unconsciousness is often transient (Gibson et al., 2016). Incomplete severance of the carotid (and possibly vertebral) arteries may maintain brain perfusion, resulting in a slow or incomplete death (Gregory and Wotton, 1986). These outcomes would raise serious animal welfare concerns and are not compliant with legal standards (e.g., Council Regulation [EC] No 1099/2009). To ensure humane slaughter, stun-to-stick intervals should be minimized, ideally to 20 seconds after stunning in order to prevent recovery of consciousness before exsanguination (Verhoeven et al., 2015). This time was not exceeded in our study. Accurate and timely neck cutting, along with species- and system-specific adjustments of stunning parameters, are essential to meet ethical and legal requirements.

### Influence of subgroups

The results suggest that different fattening methods are associated with variations in broiler responses throughout the slaughter process. Broilers from LIT and HEV showed more welfare-relevant behaviors in the pre-stunning phase, such as wing flapping during shackling. In contrast, ORG broilers were more likely to show signs of inadequate stunning, including a higher incidence of pupillary reflexes and body movements. Our analysis indicates that fattening methods differ in their suitability for specific phases of the slaughter process, possibly owing to differences in body size uniformity, fattening environment, or muscle and fat distribution. In addition to fattening methods effects, animal age emerged as a key influencing factor across multiple stunning-related parameters. With increasing age, the risk of wing flapping and inadequate stunning decreased, pointing to a potentially greater responsiveness to electrical current or more stable unconsciousness. However, older animals also displayed higher rates of defensive behavior during shackling and an increased risk of pre-stun electric shocks. These divergent trends suggest that age-related physiological changes may influence both reactivity to handling and the physiological response to stunning. Together, the results emphasize that both fattening method and age should be considered when setting and evaluating stunning parameters. Several studies have demonstrated that different broiler breeds show distinct behavioral and physiological responses under similar fattening conditions. For example, fast‑growing breeds often show reduced activity and limited use of environmental enrichment than slower growing breeds, implying that a one‑size‑fits‑all approach may fail to account for substantial welfare-related differences between broiler types (Nicol et al., 2024). Adjustments to handling and stunning procedures, particularly for older or heavier animals, may be necessary to minimize stress and ensure humane slaughter conditions across all age groups. Tailored stunning protocols that account for fattening methods, age, and body composition could improve animal welfare outcomes and ensure compliance with legal and ethical standards.

Beyond fattening method and age, several other variables were examined for their potential influence on the effectiveness of EWB stunning. Among these, transport time was not significantly associated with indicators of stunning effectiveness in our study. This finding may indicate that the animals had sufficient time to rest after transport — not too short, but also not excessively long — allowing them to partially recover from pre-transport stress, which is beneficial for their welfare prior to being stunned. According to Vieira et al. (2024), the waiting time in lairage should be kept short (1 to 2 hours) to minimize stress and maintain animal welfare. The duration of stay in the lairage area was only recorded sporadically in our study and therefore could not be included as a consistent variable in the analysis. In contrast, transport duration and environmental stressors are known to play a more critical role in controlled atmosphere stunning methods, where animals remain in their crates. The literature shows that prolonged transport under suboptimal thermal conditions can exacerbate physiological stress and negatively affect welfare outcomes in controlled atmosphere stunning systems (Terlow et al., 2008). Although transport time was not associated with EWB stunning outcomes in this study, it remains an important parameter in slaughterhouse logistics when comparing stunning methods. In contrast, carcass weight was significantly associated with behavior during the shackling process. In both years, higher carcass weight was associated with a reduced risk of wing flapping during shackling. This may be due to reduced mobility or reactivity in heavier animals, highlighting the potential role of physical characteristics in shaping behavioral responses during handling. Due to data limitations, particularly the lack of seasonal variation, weather conditions could not be comprehensively evaluated. As a result, their potential influence on stunning outcomes remains unclear in this study. This relationship would be interesting for CO_2_ stunning (Gerritzen et al., 2013). Stunning parameters such as total current and voltage varied across the fattening methods and had measurable effects on post-stunning indicators. In 2021, an increase in total current was associated with a lower probability of observing rhythmic breathing, body movement, and pupillary reflexes after neck cutting. This finding supports the importance of achieving sufficient current per animal to induce immediate and irreversible unconsciousness. Interestingly, ORG broilers were often exposed to higher voltages. This result likely reflects physiological differences, as organically reared animals may show higher electrical resistance due to factors such as greater muscle mass or lower water content. To overcome this resistance, a higher voltage is required to reach the minimum current threshold necessary for effective stunning.

### Limitations

A primary limitation of this study is the lack of individual-level data, as stunning parameters and outcomes were recorded only as mean values per flock. This restriction limits the ability to precisely determine threshold values or fully account for within-flock variability. Furthermore, maintaining a consistent number of animals in the water bath proved unfeasible under commercial conditions. Gaps in the shackle line resulted in the simultaneous presence of fewer animals than intended (e.g., three or four instead of five), making it impossible to determine the exact current per animal. Consequently, stunning effectiveness was analyzed based on total current, which is considered the primary determinant of success and relates to the minimum requirements of Council Regulation (EC) No 1099/2009. Additionally, while voltage values were standardized to control for variation between fattening methods, the data originates from a single slaughterhouse, which may limit the broader generalizability of the findings. To improve the reliability of future results, it is recommended that studies record data at the individual level and maintain a continuous shackle line to ensure consistent contact quality and current delivery.

## Conclusion

This study demonstrates that improving animal welfare during EWB stunning requires optimization of key operational parameters tailored to fattening methods and animal characteristics. Variables such as current strength, water bath height, carcass weight, and electrical resistance significantly influence stunning effectiveness beyond age and fattening method alone. Although pre-stun shocks and pronounced defensive behaviors were rarely observed, their potential welfare impact highlights the importance of continuous monitoring and immediate corrective stunning when necessary. Overall, humane slaughter depends on the precise adjustment of electrical parameters and appropriate animal handling to minimize stress and ensure effective stunning. Future research should focus on developing system-specific recommendations across diverse fattening methods and comparing EWB with alternative stunning methods, such as controlled atmosphere stunning. This multifactorial approach is essential for establishing robust, system- and species-appropriate welfare standards in poultry slaughter.

In summary, these results emphasize that beyond fattening method and age, a range of animal-related and process-specific variables, such as carcass weight, electrical resistance, and current settings, can influence stunning effectiveness. Moreover, the stunning method–specific context (e.g., controlled atmosphere stunning vs. EWB stunning) should be carefully considered in welfare evaluations. Comparative research across stunning systems would be valuable to develop species- and system-appropriate welfare standards

## Funding

The project was supported by funds of the Federal Ministry of Agriculture, Food and Regional Identity (BMLEH) based on a decision of the Parliament of the Federal Republic of Germany via the Federal Office for Agriculture and Food (BLE) under the innovation support programme (Grant Number 2817804A18, Project CasStunn).

The authors extend their gratitude to all workers at the slaughterhouse for their invaluable assistance and support during the CasStunn project.

## Declaration of AI and AI-assisted technologies

The authors used DeepL SE to enhance language clarity under full human supervision. All content was reviewed, edited, and approved by the authors, who are fully responsible for the final manuscript.

## Ethical statement

All procedures involving animals were conducted in accordance with international and national guidelines for humane animal treatment and complied with relevant legislation and ethical standards, including the International Association of Veterinary Editors (IAVE) Guidelines.

All birds were slaughtered under routine commercial conditions in accordance with the German Animal Welfare Act and the Council Regulation (EC) No 1099/2009 on the protection of animals at the time of killing. The study was purely observational. Observations and data collection were performed non-invasively during routine slaughter procedures and did not interfere with the normal slaughter process or involve any additional handling or experimental intervention for research purposes. No personal or identifiable human data were collected. According to institutional and national regulations, formal ethical review and approval were therefore not required for this study.

## CRediT authorship contribution statement

**Yukari Togami:** Writing – review & editing, Writing – original draft, Visualization, Validation, Project administration, Methodology, Investigation, Formal analysis, Data curation. **Jolien Hacker:** Writing – review & editing, Writing – original draft, Visualization, Validation, Methodology, Investigation, Formal analysis, Data curation. **Elke Rauch:** Writing – review & editing, Supervision, Project administration, Methodology, Funding acquisition, Data curation, Conceptualization. **Michael Erhard:** Writing – review & editing, Supervision, Project administration, Funding acquisition. **Paul Schmidt:** Writing – review & editing, Visualization, Formal analysis. **Helen Louton:** Writing – review & editing, Visualization, Validation, Supervision, Project administration, Methodology, Funding acquisition, Conceptualization.

## Disclosures

The authors declare that they have no identifiable conflicting financial interests or personal affiliations that might have impacted the outcomes or findings of the current study.
